# Ceftazidime‐Induced Agranulocytosis: A Case Report

**DOI:** 10.1002/ccr3.71699

**Published:** 2025-12-17

**Authors:** Bingbin Dong, Xin Wu, Changbao Huang

**Affiliations:** ^1^ Department of Emergency Medicine Yijishan Hospital, First Affiliated Hospital of Wannan Medical College Wuhu China; ^2^ Department of Thyroid and Breast Surgery Yijishan Hospital, First Affiliated Hospital of Wannan Medical College Wuhu China; ^3^ Department of Gastrointestinal Surgery Yijishan Hospital, First Affiliated Hospital of Wannan Medical College Wuhu China

**Keywords:** adverse drug reaction, agranulocytosis, ceftazidime, drug safety, neutropenia

## Abstract

The most common adverse events associated with ceftazidime include hypersensitivity reactions, gastrointestinal disturbances, and transient abnormal liver function tests, whereas neutropenia is a rare complication. We present a case of ceftazidime‐induced agranulocytosis in an 89‐year‐old Chinese woman who presented with right upper quadrant abdominal pain, nausea, and vomiting. Imaging confirmed choledocholithiasis, cholecystitis, and hepatic cysts, and laboratory tests revealed elevated inflammatory markers. Treatment with intravenous ceftazidime initially improved her symptoms and inflammatory markers; however, severe neutropenia subsequently developed, progressing to agranulocytosis. After excluding other potential causes, ceftazidime was discontinued, and granulocyte colony‐stimulating factor (G‐CSF) was administered, leading to hematological recovery. This case underscores that ceftazidime, albeit rarely, can cause severe drug‐induced agranulocytosis. Clinicians should consider this possibility in cases of unexplained cytopenia, as prompt drug cessation and G‐CSF therapy may facilitate timely hematological recovery.

## Key Clinical Message

Ceftazidime, though rare, can cause life‐threatening agranulocytosis. Unexplained neutropenia during therapy should prompt immediate suspicion. Early recognition, prompt drug discontinuation, and administration of granulocyte colony‐stimulating factor are crucial and can lead to rapid hematological recovery.

## 1 Introduction

Ceftazidime is a third‐generation cephalosporin antibiotic used primarily to treat serious bacterial infections caused by aerobic Gram‐negative bacteria. It also has limited activity against certain aerobic Gram‐positive bacteria. Since its introduction in the early 1980s, ceftazidime is now frequently used to treat infected patients [1]. It is well tolerated, with a good safety profile, and the incidence of adverse reactions is low. The most common adverse events are hypersensitivity reactions, gastrointestinal events, and transient abnormal liver function tests [2]. Neutrophils are essential for host defense, phagocytizing, killing, and digesting bacteria and fungi before they multiply and cause disease. Neutropenia is one of the most common abnormalities on complete blood count encountered in clinical practice. A reduction of neutrophils in the blood to an absolute neutrophil count (ANC) of 1.5 × 10^9^ cells/L is considered neutropenia. An ANC < 0.5 × 10^9^ cells/L is considered severe neutropenia (often termed agranulocytosis), and individuals with an ANC < 0.1 × 10^9^ cells/L are at severe risk of morbidity and mortality from infections. Severe neutropenia or agranulocytosis associated with ceftazidime has been very rarely reported. We describe here a case of ceftazidime‐induced agranulocytosis.

## 2 Case History

**FIGURE 1:**
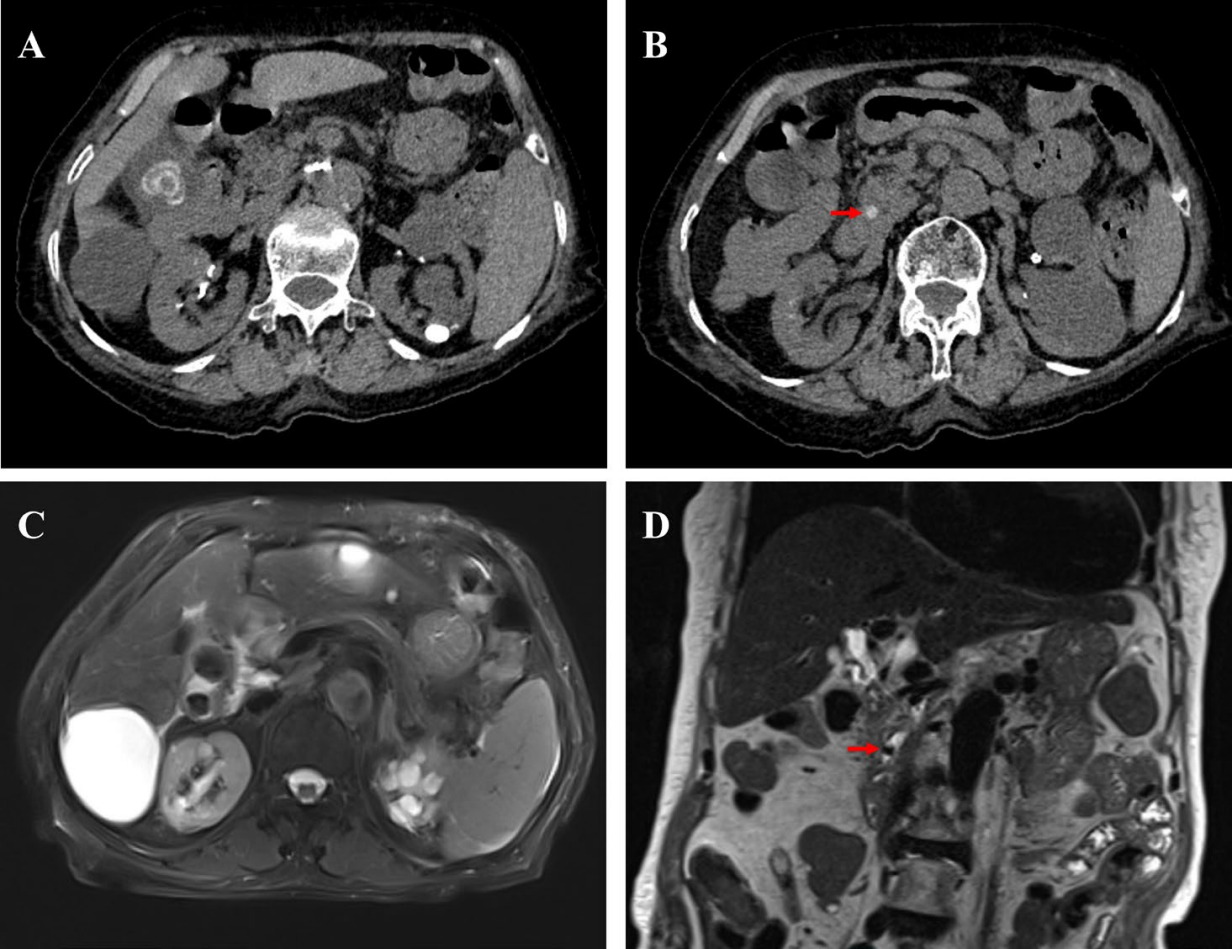
CT and MRCP features before treatment of the patient: CT scan showing gallstones and hepatic cysts (A) and a calculus in the distal common bile duct (B); MRCP scan showing gallstones and hepatic cysts (C) and a calculus in the distal common bile duct (D). Red arrow indicates the calculus in the distal common bile duct.

An 89‐year‐old Chinese woman presented to our hospital with right upper quadrant abdominal pain lasting 4 days on May 11, 2025. The patient presented with nausea and vomiting (without fever), and an emergency non‐contrast CT of the entire abdomen performed promptly on the day of admission revealed: a calculus in the distal common bile duct (choledocholithiasis), gallstones (cholelithiasis), and hepatic cysts (Figure 1). The patient experienced an acute ischemic stroke 1 month ago and was currently on dual antiplatelet therapy with aspirin and clopidogrel. The white blood cell count (WBC) was 15.23 × 10^9^/L, the ANC was 10.88 × 10^9^/L, Procalcitonin (PCT) was 2.52 ng/mL, C‐reactive protein (CRP) was 89.72 mg/L. However, her liver function and pancreatic function tests were within normal limits.

## 3 Methods (Differential Diagnosis, Investigations and Treatment)

Our treatment plan was NPO (nothing by mouth), antibiotic therapy, and nutritional support. We administered ceftazidime 1 g every 12 h intravenously. Following therapeutic intervention, the resolution of abdominal pain and gradual subsidence of nausea/vomiting were observed. Moreover, the inflammatory markers demonstrated a favorable response by day 4. The WBC count, ANC, and PCT all showed significant decreases. Most notably, the C‐reactive protein (CRP) level exhibited a sharp decline from 89.72 mg/L on day 0 to 25.46 mg/L. The patient underwent magnetic resonance cholangiopancreatography (MRCP) examination on May 14, 2025, day 4 post‐treatment. Imaging findings revealed multiple gallstones and cholecystitis, a stone in the distal common bile duct with associated mild extrahepatic bile duct dilatation and hepatic cysts (Figure 1). However, by day 6 post‐treatment, the patient's WBC count and ANC began to decline below the normal range, but there was no significant change in hemoglobin and platelets compared to the previous levels. Subsequently, the patient's WBC and ANC further decreased. On hospital day 9, the WBC count fell to 0.9 × 10^9^/L and the ANC to 0.2 × 10^9^/L. The patient's CRP and PCT have returned to the normal range. Hemoglobin and platelet levels remained unchanged from previous levels. Abdominal pain symptoms and abdominal signs had resolved, and the patient had started a liquid diet. Therefore, the patient was administered one daily dose of colony‐stimulating factor (CSF) via subcutaneous injection. However, the patient's WBC and ANC showed no improvement. On hospital day 11, the WBC decreased to 0.6 × 10^9^/L and the ANC became undetectable (0.0 × 10^9^/L). In the absence of evidence for infection‐induced agranulocytosis or preexisting immune disease, ceftazidime‐induced agranulocytosis was suspected. Consequently, ceftazidime was discontinued. CSF therapy was maintained.

## 4 Results (Outcome and Follow‐Up)

**FIGURE 2:**
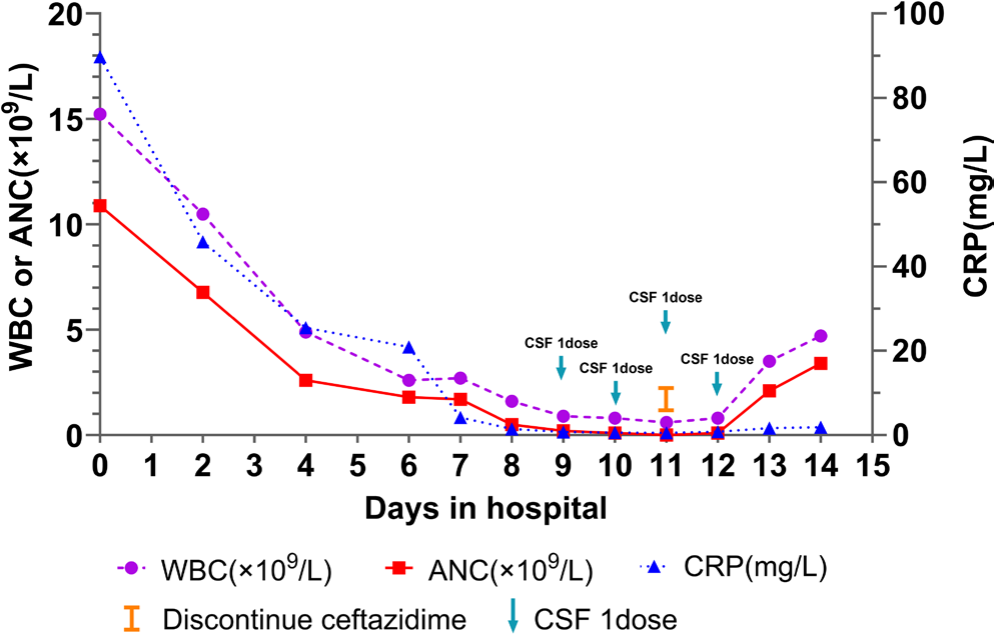
The relationship between changes in complete blood count (CBC) and C‐reactive protein (CRP) levels with the timing of administration during hospitalization. Blue arrow indicates the timing of colony‐stimulating factor administration. Orange vertical line indicates the timing of discontinuation of ceftazidime. ANC, absolute neutrophil count; CRP, C‐reactive protein; CSF, colony‐stimulating factor; WBC, white blood cell.

The patient's WBC and ANC gradually recovered. By hospital day 14, both parameters had returned to the normal range: the WBC increased to 4.7 × 10^9^/L and ANC rose to 3.4 × 10^9^/L (Figure 2). Following multidisciplinary consultation and informed consent, endoscopic retrograde cholangiopancreatography (ERCP) was recommended for postponement due to significantly elevated risks in this elderly patient with recent stroke on dual antiplatelet therapy. Given resolution of symptoms, normalized infection markers, and resumption of regular diet, the patient was discharged on hospital day 15. At the one‐month follow‐up visit, the patient remained free of abdominal pain, fever, or other symptoms, with WBC, ANC, and CRP all within normal ranges.

## 5 Discussion

This is an uncommon case of adverse drug response caused by ceftazidime. Although mentioned in the drug prescribing information, reports of ceftazidime‐induced agranulocytosis have been exceptionally rare in the literature. It is noteworthy that a large population‐based surveillance study has confirmed the association of agranulocytosis with β‐lactam antibiotics (which include cephalosporins), despite an overall low attributable incidence of approximately 0.42 per million per year [3].

In our case, the patient was diagnosed with a biliary tract infection caused by biliary tract stones including choledocholithiasis and gallstones. Choledocholithiasis refers to the presence of calculi within the common bile duct. Choledocholithiasis is a common presentation of symptomatic cholelithiasis that can result in biliary obstruction, cholangitis, and pancreatitis. Choledocholithiasis may present as right upper quadrant pain that is more prolonged than typical episodes of biliary colic; as symptoms of obstructive jaundice such as dark urine, scleral icterus, and acholic stools; or as ascending cholangitis with Charcot's triad of fever, right upper quadrant pain, and jaundice [4]. Gallstones are one of the most common of all the gastrointestinal diseases. Most gallstones patients are asymptomatic. Symptomatic gallstones patients typically present as steady, moderate to severe epigastrium or right upper quadrant of the abdomen pain lasting several hours [5]. In this case, the patient presented with right upper quadrant pain, nausea, and vomiting, without fever or jaundice. Moreover, her inflammatory markers were significantly elevated. Both abdominal CT and MRCP revealed choledocholithiasis and gallstones in the patient. Therefore, the diagnosis of biliary tract stones and infection is confirmed. After treatment with ceftazidime, the patient's symptoms, signs, and inflammatory markers improved. However, subsequent neutropenia developed.

Drug‐induced neutropenia results from either impaired neutrophil production or accelerated neutrophil destruction. Neutropenia includes chemotherapy‐induced neutropenia (CIN) and non‐chemotherapy idiosyncratic drug‐induced neutropenia (IDIN). IDIN is a relatively rare but potentially fatal disorder that occurs in susceptible individuals [6]. Acute neutropenia is most commonly associated with infections and drugs. In our case, the patient was diagnosed with a biliary tract infection and treated with ceftazidime. She demonstrated a decreased absolute neutrophil count without significant fever or other clinical manifestations suggestive of infection. Moreover, inflammatory markers PCT and CRP showed rapid and sustained improvement, with CRP normalizing to 4.16 mg/L by day 7. However, the neutrophil count began its decline to neutropenic levels precisely as the infection was resolving. This inverse temporal relationship indicated that the neutropenia was not due to infection but was associated with the drug. Anti‐infective agents have been identified as a common cause of drug‐induced neutropenia based on epidemiological studies [7]. Thus, ceftazidime was suspected to be the etiology of agranulocytosis. We promptly discontinued the drug and initiated granulocyte colony‐stimulating factor (G‐CSF) therapy, resulting in rapid hematopoietic recovery. This clinical course further substantiates ceftazidime as the causative agent of drug‐induced agranulocytosis.

In many cases, agranulocytosis typically has a delayed presentation and may pose a life‐threatening risk. Preventive measures are therefore required, particularly for drugs carrying significant agranulocytosis risk. Before starting treatment, review all medications and monitor blood counts closely. Immediately discontinue any drugs that may trigger agranulocytosis and initiate G‐CSF therapy [8].

In conclusion, this case underscores ceftazidime as an infrequent yet clinically significant cause of drug‐induced agranulocytosis. Although rare, this adverse effect carries substantial risk due to its potential severity, including life‐threatening infections secondary to agranulocytosis. Clinicians should maintain a high index of suspicion for ceftazidime‐associated agranulocytosis when evaluating patients with unexplained cytopenias, particularly those receiving prolonged or high‐dose therapy. Critically, prompt recognition followed by immediate discontinuation of the offending agent and administration of colony‐stimulating factors (CSF) can facilitate rapid hematological recovery and mitigate complications.

## Author Contributions

**Bingbin Dong:** conceptualization, data curation, investigation, visualization, writing – original draft, writing – review and editing. **Xin Wu:** conceptualization, investigation, methodology, writing – review and editing. **Changbao Huang:** data curation, project administration, visualization.

## Funding

This work was supported by General Surgery National Clinical Key Specialty Construction Funds (Finance KGZ002).

## Disclosure

The authors have nothing to report.

## Ethics Statement

The authors have nothing to report.

## Consent

Informed written consent was obtained from the patient prior to the drafting of the case report.

## Conflicts of Interest

The authors declare no conflicts of interest.

## Data Availability

The data that support the findings of this study are available in the study itself.
